# Are socio-economic inequalities related to cardiovascular disease risk? A systematic review and meta-analysis of prospective studies

**DOI:** 10.1186/s12872-024-04248-5

**Published:** 2024-11-27

**Authors:** Ololade J. Baruwa, Federica Alberti, Sunday Onagbiye, Annalisa Guddemi, Anna Odone, Hannah Ricci, Maddalena Gaeta, Schmid Daniela, Cristian Ricci

**Affiliations:** 1 Africa Unit for Transdisciplinary Health Research (AUTHeR), North-West University, Potchefstroom, South Africa; 2 University of Cape Town, Cape Town, South Africa; 3 Department of Public Health, Experimental and Forensic Medicine, University of Pavia, Pavia, Italy; 4 Health & Exercise Science, Frederick Community College, Frederick, MD USA; 5 Faculty of Life Sciences, Albstadt-Sigmaringen University, Sigmaringen, Germany

**Keywords:** Socio-economic inequality, Cardiovascular disease, Meta-analysis

## Abstract

**Purpose:**

The aim of this research was to investigate the relationship between socio-economic inequalities and fatal and non-fatal cardiovascular events.

**Methods:**

A systematic review of recently published cohort studies and a meta-analysis of relative risk (RR) of low compared with high socio-economic status (SES) in relation to cardiovascular incidence and mortality was conducted. Supplementary evaluations were conducted considering different proxies of SES in relation to different types of cardiovascular disease (CVD).

**Results:**

We identified 17 studies including approximately 26.5 million of participants with more than 900,000 CVD events. We estimated a 50% increased CVD risk for low SES with respect to high SES (RR = 1.49 [95% confidence interval: 1.26, 1.78]). For sex-specific risk, we estimated a 79% increased CVD risk for women of low SES (RR = 1.79 [1.30, 2.46]). In men, the same investigation found a 45% increased CVD risk (RR = 1.45 [1.09, 1.92]). We reported that low education (RR = 1.56 [1.27, 1.91]), increased CVD risk the most, more than low income (RR = 1.38 [1.12, 1.70]).

**Conclusion:**

Although not statistically significant, women of low SES were at higher CVD risk than men. CVD risk was more relevant to educational inequality than economic inequality.

**Supplementary Information:**

The online version contains supplementary material available at 10.1186/s12872-024-04248-5.

## Introduction

Cardiovascular diseases (CVDs), including stroke, heart attack, coronary heart disease and other cardiovascular conditions, are the leading cause of mortality and reduced quality of life worldwide [1]. The burden of CVDs has continued to increase in the last three decades. In 1990, overall CVD prevalence and mortality was estimated at 270 million and 12 million, respectively [1, 2]. In 2019, these numbers had nearly doubled with an observed prevalence of 523 million and 17.9 million for CVD incidence and mortality, respectively. Of these deaths, 85% were reportedly attributed to stroke and heart attack [1].

CVD represents a universal threat to global health. In addition, the current rise in socio-economic inequality is worrying, with increased income inequality reported worldwide in the last three decades [3]. In the last 20 years, the earnings of the richest individuals has risen globally while the poorest have seen their economic well-being diminish [4, 5]. More precisely, despite economic progress from 1990 to 2015, income going to the richest 1% of the population increased in 59 out of 100 countries; at the same time, the poorest 40% earned less than 25% of all income in 92 out of 100 countries [3]. Rising economic inequality is only one aspect of increasing socio-economic inequality [3, 6, 7] and the very nature and measurement of socio-economic inequality is a matter of controversy and discussion. An individual’s socio-economic status (SES) is a commonly used and convenient proxy for socio-economic inequality in a given population [7]. The global CVD pandemic and the global rise of economic and social inequalities are not independent threats. Scientific research has shown that individuals of lower SES have increased risk, suffer more, and die faster and more often from CVD compared with individuals of higher SES [7–10]. Potential mechanisms of action have been proposed to explain the way on which low SES may influence health. Among these, less favourable demographic, epidemiological, and nutritional conditions were identified early as possible underlying factors in the relationship between low SES and higher CVD risk [9, 11–14]. In addition, poor health care access and a lack of effectiveness of CVD prevention programmes increase CVD risk in people of low SES [8, 14, 15].

Few systematic reviews and meta-analyses have been conducted to investigate the magnitude of low SES in relation to CVD risk. Briefly, a 20–40% increased CVD risk has been observed for various CVD outcomes [10], while a more modest 15% increased risk was reported for metabolic syndrome in relation to low SES [16]. Much of the available evidence is not particularly current, having stemmed from studies published before 2019. This is possibly because of practical and methodological challenges; in particular, the best method of quantifying SES remains unclear. Terms such as social class, social stratification and social status are often used interchangeably to describe SES despite their different theoretical bases and interpretations [17].

Investigating the association between SES and CVD risk is not without controversy. However, such investigation remains essential to appropriately tailoring strategies to mitigate the consequences of socio-economic disparities to CVD risk. To this aim, we conducted a meta-analysis and systematic review of recently published prospective studies investigating the relationships between SES, overall CVD and specific CVD risk. Different types of SES measurement were considered, to better understand the way in which SES influences the CVD risk. Furthermore, we used this meta-analysis to investigate potential determinants of the heterogeneity observed in the relationship between SES and CVD risk. Geographical factors, year of publication and methodological quality were also considered in our investigation of socio-economic inequality and CVD risk.

## Methods

### Eligibility criteria

The reporting of this systematic review and meta-analysis was conducted according to the Preferred Reporting Items for Systematic Reviews and Meta-Analyses (PRISMA) [18]. Paper selection was defined by the Population, Exposure, Comparator, Outcome (PECO) approach, an adaptation of the Population, Intervention, Comparator, Outcome (PICO) criteria for systematic reviews of intervention studies [19, 20]. A time factor and a constraint to the study design were introduced, as we considered prospective studies published after the 1 January 2012 to enhance homogeneity between studies and to base our work on the most recent evidence available. Studies were excluded if the full article was not published in the English language. Systematic reviews and meta-analyses, reports, patents, theses, posters, conference reports, letters, opinion papers and seminar papers were also excluded. The literature research was computed using Medical Subject Headings (MeSH) according to the PECO (ST) criteria, where the S and T items represent the study design (S) and the timing of publication (T). The OR and NOT operators were used to link the MeSH search terms within the PECO (ST) items, and the AND operator was used to link search strings from single PECO (ST) items. As a first step, potentially eligible papers were identified after examination of the titles and abstracts by two independent reviewers. Afterwards, the same two co-authors independently proofread the full-text articles of those potentially eligible studies. Any disagreement about study inclusion was solved by discussion or by consulting a senior author. The overall study quality and risk of bias was assessed by two independent co-authors using the Newcastle–Ottawa Scale (NOS) [21], a validated tool developed to assess potential bias and overall quality in observational studies included in meta-analyses.

### Statistical analyses

The main statistical analysis was a meta-analysis of relative risk (RR) estimates from a multivariate adjusted regression evaluating the risk of fatal and non-fatal CVD outcome for low SES compared with high SES. Briefly, the meta-analysis of RRs for the lowest category with respect to the highest one is a common practice and an attempt at homogeneous differential of exposures among different studies [22]. This analysis was conducted using a random effect approach with inverse variance weighting. The weight of the i-th study was computed as w_i_ = 1/(s_i_^2^ + t^2^), where s_i_^2^ was the variance estimate from the i-th study, and t^2^ was the overall variance. The fixed effect estimate was also reported for illustrative purposes. In our meta-analysis, all studies contributed with a single RR only. A fixed effect meta-analysis was conducted to provide a single RR when more than one RR was reported by a study (for example, if RRs were reported by sex or by more than one type of CVD, separately). Furthermore, to avoid possible residual confounding, only the RR from the fully adjusted model was considered if more than one result was reported considering different adjusting covariates.

Between-study heterogeneity was reported using the I^2^ statistic (relevant between-study heterogeneity was defined by I^2^ > 50%) and statistically significant between-study heterogeneity was assessed using the Cochrane Q test. Stratification and meta-regression were applied to investigate potential sources of variation. A meta-regression model was conducted with the logarithm RR as the outcome, with the above-described weight of the i-th study. The outlier effect of single studies was assessed by excluding one study at a time. Publication bias was assessed using Egger’s test; if less than nine estimates were meta-analysed, visual inspection of the funnel plot was also used [23].

The statistical analyses were conducted using STATA version 14 and all statistical tests were two tailed, with a type-I error rate of 5% (α = 0.05). The influence analyses, assessment of publication bias, funnel plot and meta-regression were performed using the METAN and METANINF, METABIAS, METAFUNNEL, and METAREG functions, respectively.

## Results

**Fig. 1:**
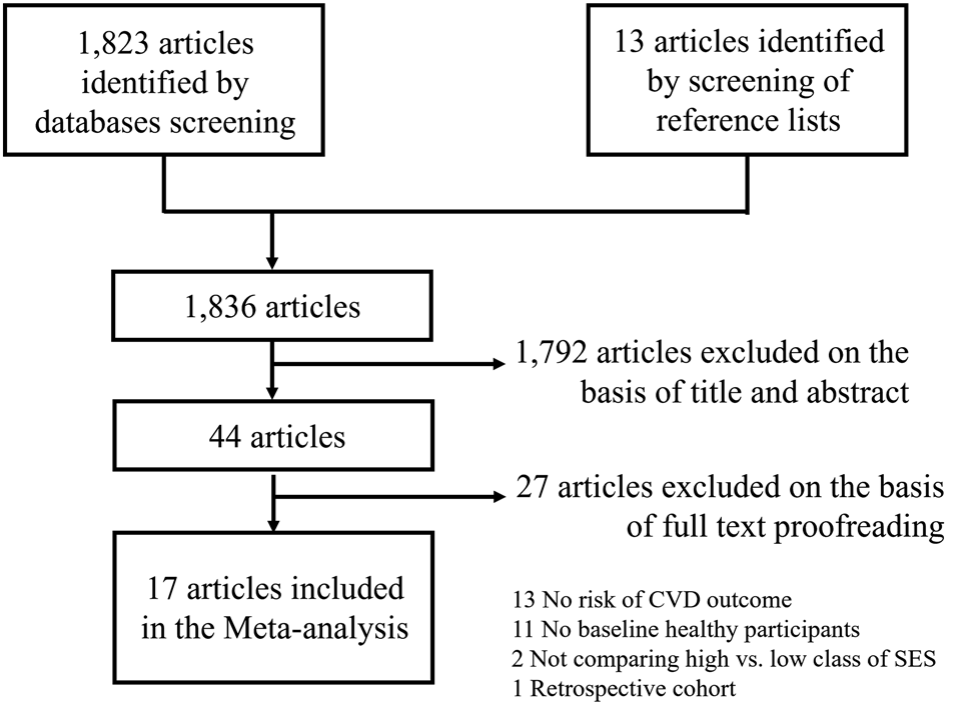
Flowchart of article selection

The selection of eligible articles is illustrated in Fig. 1.

After exclusion of duplicates from independent databases, the search identified 1823 articles, with 13 articles identified after screening the reference list of previously published systematic reviews and meta-analyses. After a first screening of the titles and abstracts, 44 articles remained for full review, of which 17 articles fulfilled the inclusion criteria [24–40]. The characteristics of the included studies are reported in Table 1. Most of the included studies were from European countries, with two studies each from Denmark and The Netherlands, and one study each from Greece, Finland and Sweden. One study was based on a prospective cohort conducted in numerous European countries (Denmark, Germany, Greece, Italy, Norway, Spain, Sweden, and the UK). The USA and Asia contributed four studies each (in Asia two studies were from China, and one study each was from South Korea and Iran). Finally, one study was from Australia.

The median age of the participants was 54 years (range = 45.5–71.9). A total of 26,435,101 participants with a total number of person-years of 291,519,169 were included in the studies. The total number of fatal and non-fatal CVD cases in the included studies was 911,791. Of these, 446,130 were myocardial infarction, 246,845 were coronary heart disease, 210,944 were strokes, 2004 were ischaemic heart disease, 1794 were atrial fibrillation and 804 were cerebrovascular disease. Four of the included studies were based on national registries [24–26, 31]. The NOS scale score ranged between 4 and 9 (median = 8). The characteristics of the included studies are reported in Table 1.

**Table 1:** Characteristics of included studies

First author, year country study design–Cohort name	Average age (years) % women	Total sample size Person-years Cases	Adjusting factors	Group, Exposure outcome, RR (95% CI)	NOS
Agyemang et al. [24] The Netherlands RGN	47.6 50.0%	Total: 2,591,170 Pers-year: 20,584,569 MI: 70,554	AGE, MAR, GEN, URB, CHR	Women, Income MI, 2.34 (2.25, 2.44) Men, Income MI, 2.07 (2.02, 2.12) All, Income MI, 2.14 (2.09, 2.18)	8
Andersen et al. [25] Denmark RGN	71.9 47.5%	Total: 2,613,045 Pers-year: 23,517,408 STRi: 50,048	AGE, SEX, YER	All, Income STRi, 1.18 (1.15, 1.22) All, Education STRi, 1.79 (1.72, 1.85)	7
Forsberg et al.[26] Sweden RGN	54.4 50.0%	Total: 3,644,309 Pers-year: 43,731,708 CHD: 246,036 STRi: 159,579	AGE, MAR, INC, EDU	Women:, nSES CHD, 1.09 (1.08, 1.10) Men, nSES CHD, 1.05 (1.04, 1.06) All, nSES CHD, 1.07 (1.06, 1.08) Women, nSES STRi, 1.04 (1.03, 1.05) Men, nSES STRi, 1.03 (1.02, 1.04) All, nSES STRi, 1.04 (1.03, 1.05)	7
)Gallo et al. [27] Europe PC–EPIC	52.8 54.7%	Total: 371,295 Pers-year: 3,166,340 CVD*: 4151 IHD*: 2004 CRB*: 855	AGE, CNR, SMK, BMI, ALC, PAC, FRV	Women, RII CVD*, 1.61 (1.28, 2.04) Men, RII CVD*, 1.53 (1.32, 1.79) All, RII CVD*, 1.56 (1.37, 1.77) Women, RII IHD*, 1.64 (1.11, 2.44) Men, RII IHD*, 1.72 (1.41, 2.17) All, RII IHD*, 1.70 (1.41, 2.06) Women, RII CRB*, 1.54 (1.01, 2.33) Men, RII CRB*-, 1.39 (0.92, 2.13) All, RII CRB*, 1.46 (1.09, 1.97)	7
Howard et al. [28] USA PC–REGARDS	64.7 55.4%	Total: 24,875 Pers-year: 186,563 STR: 929	AGE, SEX, REG, EDU, INC, SBP, HMD, SMK, HHD, DIA, LVH, ATF	Black, nSES STR, 1.01 (0.66, 1.56) White, nSES STR, 1.16 (0.85, 1.59) All, nSES STR, 1.13 (0.89, 1.42)	8
Jackson et al. [29] Australia PC-ALSWH	49.5 100%	Total: 11,468 Pers-year: – STR: 177	AGE, HOW, EDU, SMK, BMI, ALC, PAC, DEP, MAR, HYP, DIA, HHD, HYS	All, Education STR, 1.80 (0.97, 3.34)	6
Ki et al. [30] South Korea PC-KHP	53.2 53.7%	Total: 19,942 Pers-year: – CVD: 1334	AGE, SEX	All, Income CVD, 1.45 (1.04, 2.01) All, Employment CVD, 2.31 (1.10, 4.88) All, Education CVD, 1.35 (0.88, 2.09)	6
Koopman et al. [31] The Netherlands RGN	– 50.4%	Total: 15,610,000 Pers-year: 176,715,060 MI: 317,563	AGE, SEX	Women, nSES MI, 1.44 (1.42, 1.47) Men, nSES MI, 1.34 (1.32, 1.36) All, nSES MI, 1.38 (1.37, 1.40)	7
Kriegbaum et al. [32] Denmark RGN	– 51.3%	Total: 1,235,142 Pers-year: – MI: 42,669	COH, EDU, ETH	Women, Income MI, 1.78 (1.69, 1.88) Men, Income MI, 1.14 (1.10, 1.18) All, Income MI, 1.30 (1.27, 1.34) Women, Education MI, 1.61 (1.52, 1.70) Men, Education MI, 1.32 (1.28, 1.37) All, Education MI, 1.39 (1.35, 1.43) Women, APDMEI MI, 1.86 (1.77, 1.96) Men, APDMEI MI, 4.03 (3.84, 4.22) All, APDMEI MI, 2.82 (2.72, 2.92)	8
Lammintausta et al. [33] Finland RGN	56.7 55.1%	Total: 233,287 Pers-year: 23,099,583 MI: 15,374	REG, YER	Women, Income MI, 4.68 (3.40, 6.44) Men, Income MI, 2.74 (2.38, 3.16) All, Income MI, 2.99 (2.63, 3.41)	7
Lewis et al. [34] USA PC–REGARDS	64.1 57.0%	Total: 24,461 Pers-year: 146,766 CHD: 809	AGE, SEX, ETN, REG, SMK, PAC, ALC, BMI, SBP, CHL, HDL, CRP, DIA, STA, HMD, INS, ISS, DEP	All, Income CHD, 1.08 (0.92, 1.27) All, Education CHD, 1.30 (1.09, 1.56) All, Income/Education CHD, 1.42 (1.14, 1.76)	8
Li et al. [35] China PC–HRCS	55.9 61.0%	Total: 31,162 Pers-year: – CVD: –	SEX, MAR, ALC, PAC, FRV, FAT, SES	All, Income CVD, 1.83 (1.71, 1.95) All, Education CVD, 3.71 (3.38, 4.06)	8
Masoudkabir et al. [36] Iran PC–ICS	50.6 48.7%	Total: 5375 Pers-year: 24,379 CVD: 276	AGE, SEX, SMK, BMI, HYP	All, Income CVD, 1.18 (0.81, 1.70) All, Education CVD, 0.99 (0.52, 1.89)	8
Misialek et al. [37] USA PC–ARIC	54.0 54.8%	Total: 14,352 Pers-year: 295,651 AF: 1794	AGE, SEX, ETN, REG, BMI, DIA, ALC, SMK, SBP, HMD, CVD	All, Income AF, 1.02 (0.88, 1.17) Women, Education AF, 1.29 (1.06, 1.58) Men, Education AF, 0.88 (0.74, 1.05) All, Education A, 1.04 (0.91, 1.19)	9
Panagiotakos et al. [38] Greece PC–ATTICA	45.5 50.2%	Total: 2020 Pers-year: 20,200 CVD: 317	AGE, SEX, BMI, SMK, HYP, CHL, DIA, PAC, DIET, ALC	All, Education CVD, 1.44 (0.94, 2.20)	6
Senan et al. [39] USA PC–no name	47.5 24.9%	Total: 346 Pers-year: 2422 CVD: –	AGE, EDU, INC, PAC, BPS, ETH, SMK < ALC, DIA, CHL, HRT, MEP	All, Income CVD, 1.08 (0.96, 1.19) All, Education CVD, 1.37 (1.22–1.52)	4
Zhou et al. [40] China PC–Anhui cohort	71.7 51.8%	Total: 2852 Pers-year: 28,520 STR: 211	AGE, SEX, BMI, SMK, ALC, MAR, VCH, HYP, CVD, DIA, PAC, DEP, RUR, EDU, INC	Women, Income STR, 0.62 (0.30, 1.28) Men, Income STR, 0.83 (0.40, 1.73) All, Income STR, 0.72 (0.43, 1.20) Women, Education STR, 3.68 (1.70, 7.97) Men, Education STR, 0.93 (0.44, 1.98) All, Education STR, 1.82 (1.06, 3.11)	8

*Abbreviations*: RGN: National register, PC: Prospective cohort, RR: Relative risk estimate

*List of exposures*: APDMEI: Accumulated proportional deviation from median equivalised income, nSES: Neighbourhood socio economic status, RII: Relative index of inequality

*List of outcomes*: AF: Atrial fibrillation, CHD: Coronary heart disease, CRB: Cerebrovascular disease, CVD: Any cardiovascular outcome, IHD: Ischaemic heart disease, MI: Myocardial Infarction, STR: Stroke, STRi: Ischaemic stroke, *: CVD mortality cause

*List of adjusting factors*: ALC: Alcohol consumption, ATF: Atrial fibrillation, BMI: Body mass index, BPS: Blood pressure, CHL: Total cholesterol, CHR: Charlson index, CNR: Centre of recruitment, COH: Birth cohort, CRP: C-reactive protein, CVD: baseline CVD, DIA: Type II diabetes, DEP: Depression, EDU: Education, ETH Ethnicity, FAT: Dietary fat intake, FRV: Dietary intake of fruit and vegetables, GEN: Generation, HDL: High-density lipoprotein cholesterol, HHD: History of heart disease, HMD: Anti-hypertensive medications, HOW: Home ownership, HRT: Hormone replace therapy, HYP: Hypertension, HYS: Hysterectomy, INC: Income, INS: Insulin use, ISS: Health insurance status, LDL: Low-density lipoprotein cholesterol, LVH: Left ventricular hypertrophy, MAR: Marital status, MEP: Menopausal status, PAC: Physical activity, REG: Region, RUR: Rural area, SBP: Systolic blood pressure, SES: Socio-economic status, SMK: Smoking status, STA: Statins use, TRI: Triglycerides, URB: Urbanisation, VCH: Visiting of children, YER: Calendar year

### Meta-analysis of low compared with high category of socio-economic level

**Fig. 2:**
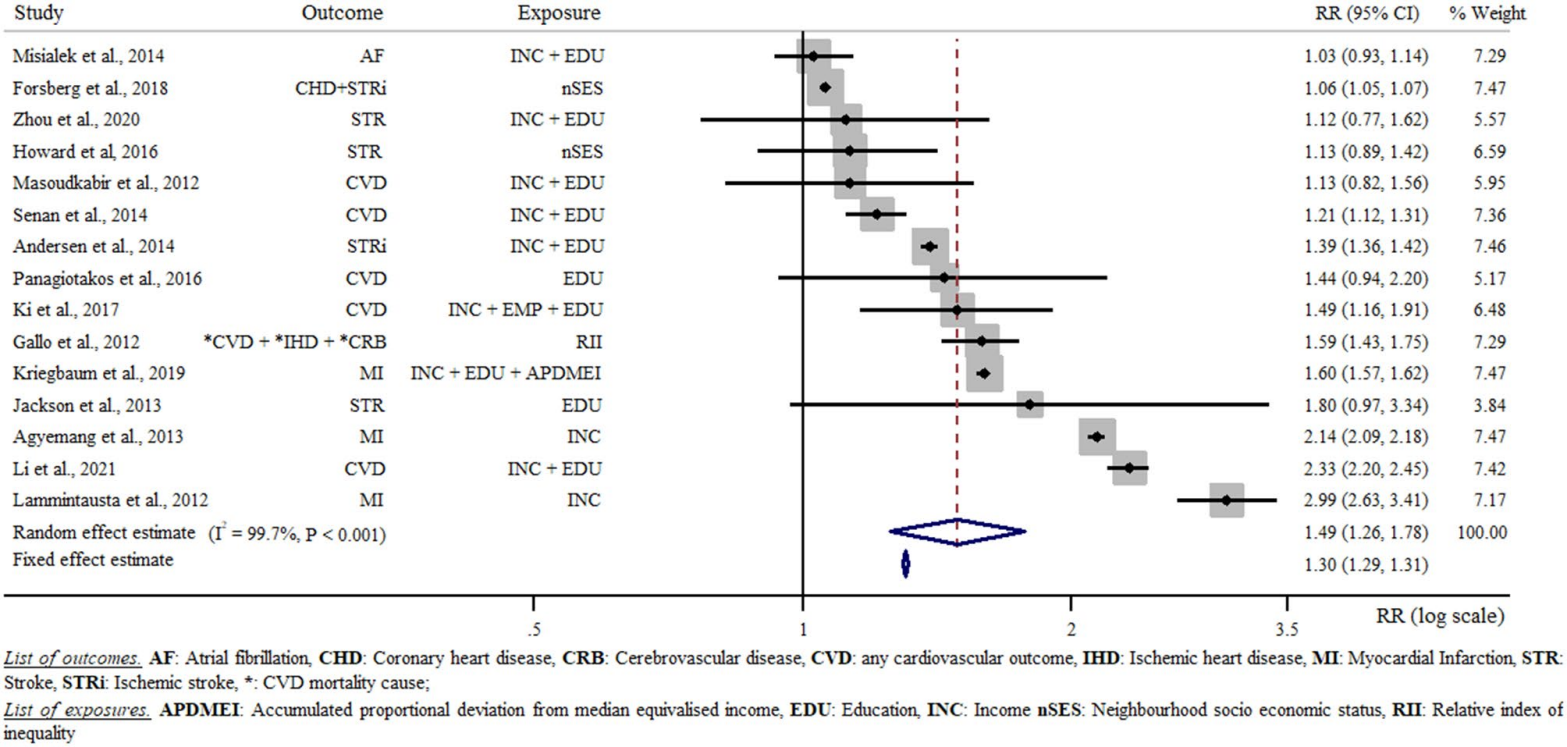
Random effect meta-analysis comparing low with high socio-economic status in relation to cardiovascular disease risk

The results of the initial meta-analysis, which was based on 15 studies [24–30, 32, 33, 35–40], and included both women and men and all exposures and all outcomes is reported in Fig. 2. This meta-analysis resulted in a cumulative sample size of 10,800,640 participants with a cumulative 114,657,343 person-years and 433,870 CVD cases. The random effect model estimated a 49% increased CVD risk for low SES compared with high SES category (RR = 1.49 [95% confidence interval: 1.26, 1.76]). Between-study heterogeneity was considerable, with single RR estimates ranging between 1.03 (0.93, 1.14) [37] and 2.99 (2.63, 3.41) [33]. The meta-analysis of low compared with high SES in relation to CVD risk is reported in Fig. 2.

**Fig. 3:**
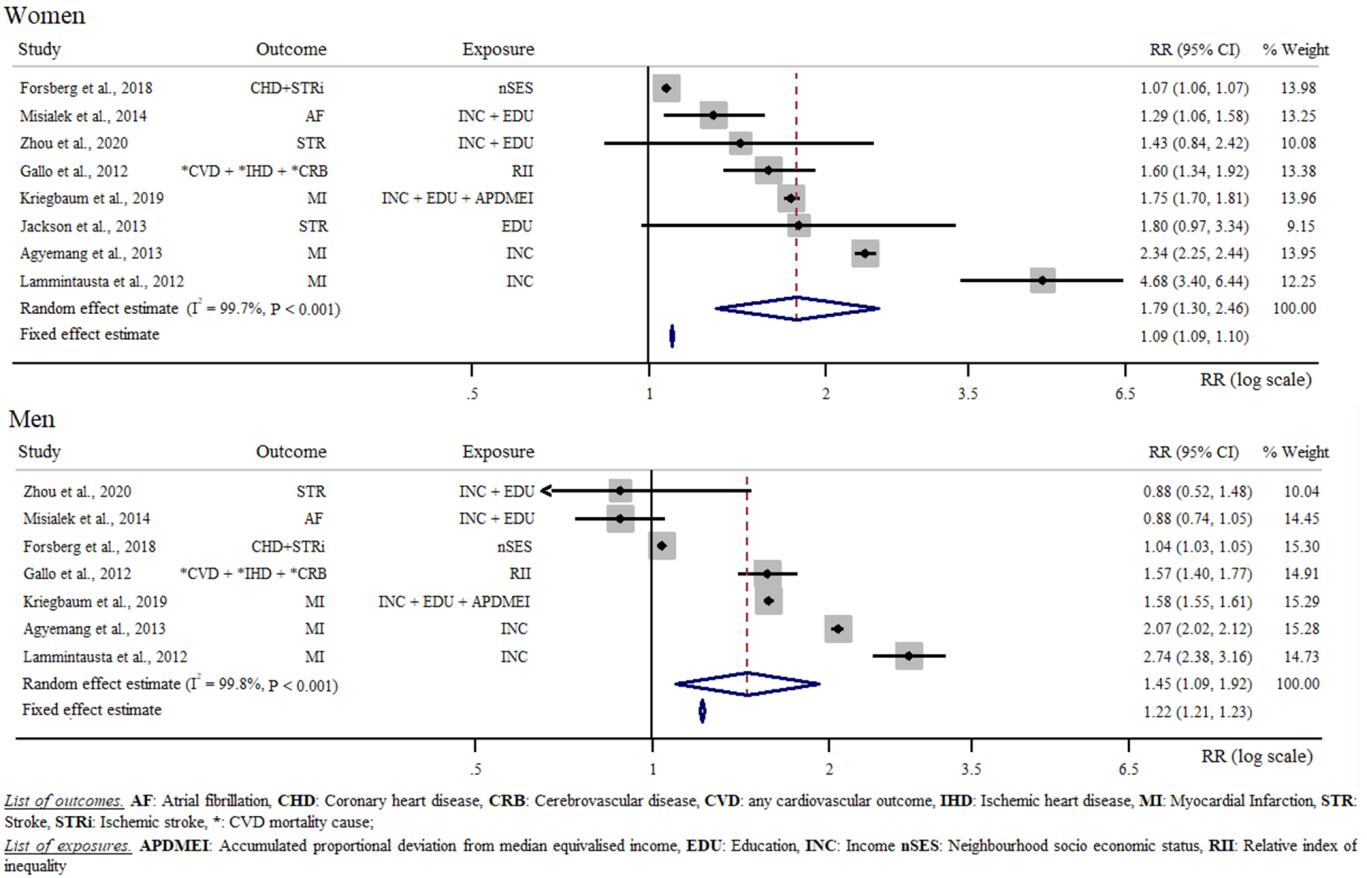
Random effect meta-analysis comparing low with high socio-economic status in relation to cardiovascular disease risk by sex

This same analysis conducted by sex revealed that women in the low SES category had higher CVD risk (RR = 1.79 [1.30, 2.46]) than men. Using a random effect meta-analysis we estimated in women a 79% increased CVD risk (RR = 1.79 [1.30, 2.46]) for low compared with high SES category. In men, the same meta-analysis revealed that men in the lowest SES class had 45% increased CVD risk when compared with their counterparts in the highest SES class (RR = 1.45 [1.09, 1.92]). Sex-stratified analyses of low SES in relation to CVD risk are reported in Fig. 3.

**Fig. 4:**
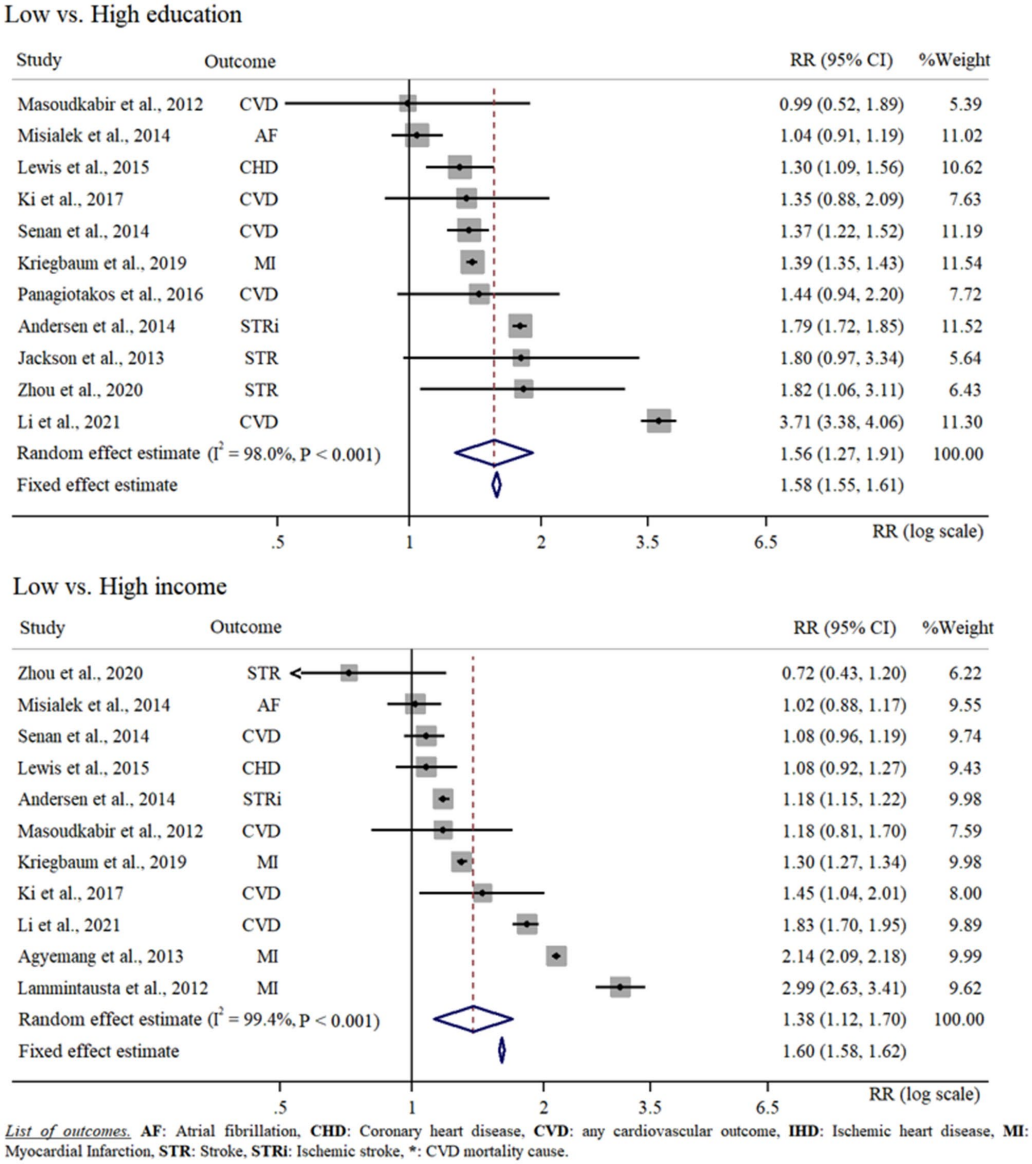
Random effect meta-analysis comparing low with high education status and low with high income in relation to CVD risk

Seven studies [24, 26, 27, 32, 33, 37, 40] provided data on SES in relation to CVD risk in both men and women. Here we observed a 12% increased CVD risk for women of low SES category with respect to men of the same SES (RR = 1.12 [1.04, 1.20]). Different SES categories were associated with different burdens of CVD risk (Fig. 4). On the one hand, the meta-analysis conducted considering education status revealed a 56% increased CVD risk for low education compared with high education (RR = 1.56 [1.27, 1.91]). On the other hand, the meta-analysis conducted considering income showed a 38% increased CVD risk when comparing low with high income categories (RR = 1.38 [1.12, 1.70]). Nine studies provided data for a random effect meta-analysis of the difference in low compared with high education level and low compared with high income [25, 30, 32, 34–37, 39, 40]. Here, we found a 30% increased CVD risk when comparing the CVD risk of low compared with high education level to the CVD risk of low compared with high income status (RR = 1.30 [1.07, 1.58]). Three studies [26, 28, 31] investigated CVD risk in relation to the SES of the neighbourhood. According to our calculation, living in a low SES neighbourhood did not result in a statistically significant CVD risk (RR = 1.19 [0.95, 1.48]). Two studies reported the CVD risk for low compared with high composite score [27, 32]. A random effect meta-analysis showed a twofold increased CVD risk for low SES category compared with high SES category (RR = 2.12 [1.21, 3.72]). Four studies specifically investigated the association between SES and stroke risk [25, 28, 29, 40]. According to our random effect meta-analysis, being of low SES would result in a 31% increased stroke risk with respect to being of high SES (RR = 1.31 [1.13, 1.51]). Finally, based on three studies [24, 32, 33], we estimated more than a twofold increased risk of myocardial infarction when comparing low to high SES (*R* = 2.15 [1.68, 2.75]). The analyses investigating education and income in relation to CVD risk are reported in Fig. 4.

### Supplementary analyses

The stratified random effect meta-analyses and the results from the meta-regressions are shown in Table 2. Age, publication year, the number of adjusting factors and the type of outcome did not influence the association between SES and CVD risk. A statistically significant greater CVD risk associated with SES was observed for studies with the largest sample size (*n* ≥ 24,875; RR = 1.77 [1.39, 2.26]; I^2^ = 47.2%) compared with studies with a low sample size (RR = 1.19 [1.07, 1.32]; I^2^ = 47.2%). Between-study heterogeneity was observed for the strata of studies with the lowest sample size (I^2^ = 47.2%). Studies with an NOS score below or equal to 6 showed a 32% increased CVD risk for low SES compared with high SES (RR = 1.32 [1.14, 1.53]; I^2^ = 30.5%). A 51% increased CVD risk for low SES compared with high SES for studies with an NOS score above 6 (RR = 1.51 [1.24, 1.85) was observed. Similarly, we observed a 71% increased CVD risk for low SES with respect to high SES when looking at studies from national registries (RR = 1.71 [1.29, 2.28]). As previously reported, a statistically higher risk of MI was observed with respect to non-MI CVD events (RR = 2.15 [1.68, 2.75] and RR = 1.34 [1.14, 1.58] for MI and non-MI CVD events, respectively, with P value for comparison = 0.015). A 38% increased CVD risk was observed for prospective cohorts comparing low SES with high SES (RR = 1.38 [1.07, 1.78]). When omitting each study at a time, we observed that the random effect estimate of CVD risk for SES ranged between 1.42 (1.18, 1.69) and 1.55 (1.36, 1.76) when excluding the studies from Lammintausta [33] and Forsberg [26], respectively. No outlier studies were detected with regards to sex, education or income, and other types of SES proxy. Stratified analyses and meta-regressions are reported in Table 2.

**Table 2:** Stratified analyses and meta-regression

	RR (95% CI)	I_1_^2^ (%)	I_2_^2^ (%)	P value
**Age**				
≥ 53.6 years	1.47 (1.17, 1.83)	99.6	99.4	0.832
< 53.6 years	1.53 (1.29, 1.81)	98.8		
Publication year*				
≥ 2014	1.42 (1.10, 1.82)	99.8	99.6	0.598
< 2014	1.56 (1.28, 1.96)	99.3		
Sample size*				
≥ 24,875	1.77 (1.39, 2.26)	99.9	73.4	***0.032***
< 24,875	1.19 (1.07, 1.32)	47.2		
**Newcastle Ottawa Scale**				
NOS > 6	1.51 (1.24, 1.85)	99.8	66.1	*0.078*
NOS ≤ 6	1.32 (1.14, 1.53)	30.5		
**Study design**				
National registry	1.71 (1.29, 2.28)	99.9	98.9	*0.058*
Prospective cohort	1.38 (1.07, 1.78)	97.2		
**Region**				
Europe	1.34 (0.98, 1.85)	97.8	85.4	0.210
Asian-Pacific	1.52 (1.05, 2.22)	90.8		
USA	1.12 (1.00, 1.27)	66.9		
**Adjusting factors**				
≥ 6	1.40 (1.04, 1.87)	97.8	99.8	0.448
< 6	1.60 (1.25, 2.04)	99.9		
**Outcome**				
Considering all CVDs	1.50 (1.11, 2.04)	97.6	99.7	0.939
Specific CVDs	1.48 (1.19, 1.85)	99.8		
Stroke	1.22 (1.00, 1.49)	99.2	99.1	0.140
No stroke	1.64 (1.40, 1.91)	98.9		
MI	2.15 (1.68, 2.75)	99.6	99.3	***0.015***
No MI	1.34 (1.14, 1.58)	99.1		

*Stratification performed by median number, I_1_^2^: heterogeneity within stratification, I_2_^2^: residual heterogeneity after meta-regression, Adj R^2^: adjusted R-squared, P value from regression omnibus statistical test

CVD: cardiovascular disease; MI: myocardial infarction; NOS: Newcastle–Ottawa Scale

## Discussion

This study confirms and reinforces results from a previous meta-analysis conducted to investigate income and education in relation to CVD risk [10]. We observed that socio-economic inequalities result in about 50% difference in CVD risk. We acknowledge that this is a rough estimate as it was calculated irrespective of sex, type of SES measure and type of CVD outcome. However, this result portrays the burden of socio-economic inequalities on CVD risk. Moreover, according to our results, socio-economic inequalities affect CVD risk in women more than in men. Even if not strictly statistically significant, increased CVD risk owing to socio-economic inequalities appears to be more relevant in women than men from a public health viewpoint. This is expected, as we consistently observed a higher CVD risk in relation to SES in women when compared with men [24, 26, 27, 32, 33, 37, 40]. This observation can thus be seen as a novel and interesting result as it explains the extent to which socio-economic inequalities affect the health of women and men [6, 41].

The most effective way to quantify socio-economic inequality and which of the possible different proxies of socio-economic inequality are the most strongly related to CVD risk remains unclear. Our study showed that education inequalities are much more strongly related to CVD risk than income inequalities. This was corroborated by seven out of the nine studies included for this aspect [25, 32, 34, 35, 37, 39, 40], for which higher CVD risk for education inequality was observed when compared with CVD risk because of income inequality. A similar result was reported by a previous meta-analysis specifically conducted in this regard [10].

In our work, we observed that socio-economic inequality according to neighbourhood SES was significantly associated with CVD risk, while socio-economic inequality measured by composite indexes resulted in a statistically significant twofold increased CVD risk. These results can be interpreted by looking at previously conducted studies. It has been suggested that low neighbourhood SES might not be associated with health risk because it does not correspond to an actual risk if neighbourhood SES is not sufficiently homogeneous [42]. Alternatively, neighbourhood SES may not influence CVD risk, as individuals in underprivileged neighbourhoods experience lower levels of stress, anxiety and relative deprivation as they do not face competition from better educated and more affluent neighbours [43]. We observed that low versus high SES measured by composite indexes such as the Relative Index of Inequality (RII) or the Accumulated Proportional Deviation from Median Equivalised Income (APDMEI) was more strongly associated with CVD risk than any other proxy of SES. This is expected for technical reasons related to the definition of these indexes. The RII is defined to correlate to health outcomes as it is empirically constructed using the parameters of linear function relating the incidence of a given outcome to SES [44]. However, the APDMEI represents an ideal measure of economic inequality, as it is a standardised measure of distance between the income of a given individual and the median income, which represents expectations. The evidence of a strong association between composite indexes and CVD is useful, as it suggests that composite indexes can be used in future studies aimed at investigating socio-economic inequalities and health.

Finally, we observed the novel result that socio-economic inequalities affect CVD risk in a specific manner, causing a twofold increased risk of myocardial infarction, a 50% increased risk of any CVD and a 30% increased risk of stroke. We can speculate that socio-economic inequalities and related factors such as poor health behaviours, reduced availability of medical treatment, psychological stress, and a lack of adequate health insurance and participation in prevention programmes act to various extents and mechanisms on specific CVD risks.

### Strengths and limitations

A strength of this study is that we based our research on prospective cohort studies, so bias can be considered negligible. Moreover, our study is of great general interest as it addresses questions that have remained open until now. These include how to effectively quantify socio-economic inequality in epidemiological studies and how socio-economic inequality may affect CVD risk differently in women compared with men. The study is also a valuable collection of potential suggestions for the design of new prospective studies investigating socio-economic inequalities in relation to health. Furthermore, our study represents a valid and novel update of the evidence linking socio-economic inequalities to CVD risk, being based on recently published research and having a strong methodological background.

Our study is not free of limitations. Firstly, we acknowledge that we did not investigate specific groups at high risk of socio-economic inequality such as people from disadvantaged or minority racial groups, the elderly and other minorities. Furthermore, the included studies were from medium to high income countries, thus excluding regions where socio-economic inequalities are more pronounced. Moreover, some of results from the stratifications performed by meta-regression could be inaccurate or overdispersed because of the merging of estimates resulting from heterogeneous definitions of SES. Finally, our stratification and meta-regression failed to address the between study heterogeneity. On the one hand, the excess of between study heterogeneity can be due to large differences between the target population and the definition of social inequalities adopted. On the other hand, the inefficiency of stratification and meta-regression to explain the between study heterogeneity is likely attributable to some studies having a very narrow confidence limits of the CVD risk estimate for high vs. low SES category. In this situation, even small differences between study risk estimates resulted in large between study heterogeneity thus nullifying the use of stratifications and meta-regressions to explain it.

## Electronic supplementary material

Below is the link to the electronic supplementary material.

Supplementary Material 1

Supplementary Material 2

Supplementary Material 3

## Data Availability

Data and analysis codes will be available by request to the corresponding author.

## Abbreviations

CVD: Cardiovascular disease
SES: Socio-economic status
RR: Relative risk
PRISMA: Preferred Reporting Items for Systematic Reviews and Meta-Analyses guidelines
PECO: Population, exposure, comparator, outcome
PICO: Population, intervention, comparator, outcome
MeSH: Medical subject headings
NOS: Newcastle Ottawa Scale

## References

[1] Roth GA, Mensah GA, Johnson CO, Addolorato G, Ammirati E, Baddour LM, et al. Global Burden of Cardiovascular diseases and Risk factors, 1990–2019: Update from the GBD 2019 study. J Am Coll Cardiol. 2020;76(25):2982–3021. 33309175 10.1016/j.jacc.2020.11.010 PMC7755038

[2] GBD 2017 Causes of Death Collaborators. Global, regional, and national age-sex-specific mortality for 282 causes of death in 195 countries and territories, 1980–2017: a systematic analysis for the global burden of Disease Study 2017. Lancet Lond Engl. 2018;392(10159):1736–88. 10.1016/S0140-6736(18)32203-7 PMC6227606 30496103

[3] United Nations. Department of Economic and Social Affairs. World social report 2020: inequality in a rapidly changing world. UN; 2020.

[4] Gwartney JD, Connors J, Montesinos H. The rise and fall of Worldwide Income Inequality. SSRN; 2018. pp. 1820–2035.

[5] Klasen S, Scholl N, Lahoti R, Ochmann S, Vollmer S. Inequality-worldwide trends and current debates. Discussion Papers; 2016.

[6] Calasanti TM, Slevin KF. Gender, social inequalities, and aging. Rowman Altamira; 2001.

[7] Lortet-Tieulent J, Saracci R, Conway DI, Straif K, Wild CP. Reducing social inequalities in cancer: evidence and priorities for research. 2019. 33443989

[8] Mannoh I, Hussien M, Commodore-Mensah Y, Michos ED. Impact of social determinants of health on cardiovascular disease prevention. Curr Opin Cardiol. 2021;36(5):572–9. 34397464 10.1097/HCO.0000000000000893

[9] Kivimäki M, Batty GD, Pentti J, Shipley MJ, Sipilä PN, Nyberg ST, et al. Association between socioeconomic status and the development of mental and physical health conditions in adulthood: a multi-cohort study. Lancet Public Health. 2020;5(3):e140–9. 32007134 10.1016/S2468-2667(19)30248-8

[10] Khaing W, Vallibhakara SA, Attia J, McEvoy M, Thakkinstian A. Effects of education and income on cardiovascular outcomes: a systematic review and meta-analysis. Eur J Prev Cardiol. 2017;24(10):1032–42. 28406328 10.1177/2047487317705916

[11] Rosengren A, Smyth A, Rangarajan S, Ramasundarahettige C, Bangdiwala SI, AlHabib KF, et al. Socioeconomic status and risk of cardiovascular disease in 20 low-income, middle-income, and high-income countries: the prospective urban rural epidemiologic (PURE) study. Lancet Glob Health. 2019;7(6):e748–60. 31028013 10.1016/S2214-109X(19)30045-2

[12] Singh-Manoux A, Fayosse A, Sabia S, Tabak A, Shipley M, Dugravot A, et al. Clinical, socioeconomic, and behavioural factors at age 50 years and risk of cardiometabolic multimorbidity and mortality: a cohort study. PLoS Med. 2018;15(5):e1002571. 29782486 10.1371/journal.pmed.1002571 PMC5962054

[13] Clark AM, DesMeules M, Luo W, Duncan AS, Wielgosz A. Socioeconomic status and cardiovascular disease: risks and implications for care. Nat Rev Cardiol. 2009;6(11):712–22. 19770848 10.1038/nrcardio.2009.163

[14] Schultz WM, Kelli HM, Lisko JC, Varghese T, Shen J, Sandesara P, et al. Socioeconomic Status and Cardiovascular outcomes: challenges and interventions. Circulation. 2018;137(20):2166–78. 29760227 10.1161/CIRCULATIONAHA.117.029652 PMC5958918

[15] Powell-Wiley TM, Baumer Y, Baah FO, Baez AS, Farmer N, Mahlobo CT, et al. Social determinants of Cardiovascular Disease. Circ Res. 2022;130(5):782–99. 35239404 10.1161/CIRCRESAHA.121.319811 PMC8893132

[16] Blanquet M, Legrand A, Pélissier A, Mourgues C. Socio-economics status and metabolic syndrome: a meta-analysis. Diabetes Metab Syndr. 2019;13(3):1805–12. 31235098 10.1016/j.dsx.2019.04.003

[17] Galobardes B, Lynch J, Smith GD. Measuring socioeconomic position in health research. 2007. 10.1093/bmb/ldm001 17284541

[18] Page MJ, McKenzie JE, Bossuyt PM, Boutron I, Hoffmann TC, Mulrow CD, et al. The PRISMA 2020 statement: an updated guideline for reporting systematic reviews. BMJ. 2021;372:n71. 33782057 10.1136/bmj.n71 PMC8005924

[19] Morgan RL, Whaley P, Thayer KA, Schünemann HJ. Identifying the PECO: a framework for formulating good questions to explore the association of environmental and other exposures with health outcomes. Environ Int. 2018;121(Pt 1):1027–31. 30166065 10.1016/j.envint.2018.07.015 PMC6908441

[20] Cumpston M, Li T, Page MJ, Chandler J, Welch VA, Higgins JP, et al. Updated guidance for trusted systematic reviews: a new edition of the Cochrane Handbook for Systematic Reviews of Interventions. Cochrane Database Syst Rev. 2019;10:ED000142. 31643080 10.1002/14651858.ED000142 PMC10284251

[21] Peterson J, Welch V, Losos M, Tugwell P. The Newcastle-Ottawa scale (NOS) for assessing the quality of nonrandomised studies in meta-analyses. Ott Ott Hosp Res Inst. 2011;2(1):1–12.

[22] Deeks JJ, Higgins JP, Altman DG, Cochrane Statistical Methods Group. Analysing data and undertaking meta-analyses. Cochrane Handb Syst Rev Interv. 2019;241–84.

[23] Sedgwick P. What is publication bias in a meta-analysis? BMJ. 2015;351:h4419. 26276792 10.1136/bmj.h4419

[24] Agyemang C, van Oeffelen Aa, Bots M, Stronks ML, Vaartjes K. Socioeconomic inequalities in acute myocardial infarction incidence in migrant groups: has the epidemic arrived? Analysis of nation-wide data. Heart Br Card Soc. 2014;100(3):239–46. 10.1136/heartjnl-2013-304721 24241713

[25] Andersen KK, Steding-Jessen M, Dalton SO, Olsen TS. Socioeconomic position and incidence of ischemic stroke in Denmark 2003–2012. A nationwide hospital-based study. J Am Heart Assoc. 2014;3(4):e000762. 25030354 10.1161/JAHA.113.000762 PMC4310360

[26] Forsberg PO, Ohlsson H, Sundquist K. Causal nature of neighborhood deprivation on individual risk of coronary heart disease or ischemic stroke: a prospective national Swedish co-relative control study in men and women. Health Place. 2018;50:1–5. 29331785 10.1016/j.healthplace.2017.12.006 PMC5834378

[27] Gallo V, Mackenbach JP, Ezzati M, Menvielle G, Kunst AE, Rohrmann S, et al. Social inequalities and mortality in Europe–results from a large multi-national cohort. PLoS ONE. 2012;7(7):e39013. 22848347 10.1371/journal.pone.0039013 PMC3405077

[28] Howard VJ, McClure LA, Kleindorfer DO, Cunningham SA, Thrift AG, Diez Roux AV, et al. Neighborhood socioeconomic index and stroke incidence in a national cohort of blacks and whites. Neurology. 2016;87(22):2340–7. 27742815 10.1212/WNL.0000000000003299 PMC5135020

[29] Jackson CA, Jones M, Mishra GD. Educational and homeownership inequalities in stroke incidence: a population-based longitudinal study of mid-aged women. Eur J Public Health. 2014;24(2):231–6. 23788011 10.1093/eurpub/ckt073

[30] Ki M, Lee YH, Kim YS, Shin JY, Lim J, Nazroo J. Socioeconomic inequalities in health in the context of multimorbidity: a Korean panel study. PLoS ONE. 2017;12(3):e0173770. 28296975 10.1371/journal.pone.0173770 PMC5351993

[31] Koopman C, van Oeffelen AA, Bots ML, Engelfriet PM, Verschuren WM, van Rossem L, et al. Neighbourhood socioeconomic inequalities in incidence of acute myocardial infarction: a cohort study quantifying age- and gender-specific differences in relative and absolute terms. BMC Public Health. 2012;12(1):617. 22870916 10.1186/1471-2458-12-617 PMC3490806

[32] Kriegbaum M, Hougaard CØ, Andersen I, Brønnum-Hansen H, Lund R. Life course analysis on income and incident AMI: a Danish register-based cohort study. J Epidemiol Community Health. 2019;73(9):810–6. 31142610 10.1136/jech-2018-212043

[33] Lammintausta A, Immonen-Räihä P, Airaksinen JKE, Torppa J, Harald K, Ketonen M, et al. Socioeconomic inequalities in the morbidity and mortality of Acute coronary events in Finland: 1988 to 2002. Ann Epidemiol. 2012;22(2):87–93. 22226031 10.1016/j.annepidem.2011.10.012

[34] Lewis MW, Khodneva Y, Redmond N, Durant RW, Judd SE, Wilkinson LL, et al. The impact of the combination of income and education on the incidence of coronary heart disease in the prospective reasons for Geographic and racial differences in stroke (REGARDS) cohort study. BMC Public Health. 2015;15(1):1312. 26715537 10.1186/s12889-015-2630-4 PMC4696109

[35] Li R, Hou J, Tu R, Liu X, Zuo T, Dong X, et al. Associations of mixture of air pollutants with estimated 10-year atherosclerotic cardiovascular disease risk modified by socio-economic status: the Henan Rural Cohort Study. Sci Total Environ. 2021;793:148542. 34174609 10.1016/j.scitotenv.2021.148542

[36] Masoudkabir F, Toghianifar N, Talaie M, Sadeghi M, Sarrafzadegan N, Mohammadifard N, et al. Socioeconomic status and incident cardiovascular disease in a developing country: findings from the Isfahan cohort study (ICS). Int J Public Health. 2012;57(3):561–8. 22314544 10.1007/s00038-012-0344-2

[37] Misialek JR, Rose KM, Everson-Rose SA, Soliman EZ, Clark CJ, Lopez FL, et al. Socioeconomic status and the incidence of Atrial Fibrillation in whites and blacks: the atherosclerosis risk in communities (ARIC) Study. J Am Heart Assoc. 2014;3(4):e001159. 25142059 10.1161/JAHA.114.001159 PMC4310413

[38] Panagiotakos D, Georgousopoulou E, Notara V, Pitaraki E, Kokkou E, Chrysohoou C, et al. Education status determines 10-year (2002–2012) survival from cardiovascular disease in Athens metropolitan area: the ATTICA study, Greece. Health Soc Care Community. 2016;24(3):334–44. 25754715 10.1111/hsc.12216

[39] Senan M, Petrosyan A. The relationship between socioeconomic status and cardiovascular events. Georgian Med News. 2014;227:42–7. 24632646

[40] Zhou W, Chen R, Hopkins A, Wang Y, Tang J, Chen X et al. Association between socioeconomic status and incident stroke in China. J Epidemiol Community Health. 2020;jech-2019-213515. 10.1136/jech-2019-213515 PMC7320795 32341052

[41] Matthews S, Manor O, Power C. Social inequalities in health: are there gender differences? Soc Sci Med 1982. 1999;48(1):49–60. 10.1016/s0277-9536(98)00288-3 10048837

[42] Hou F, Myles J. Neighbourhood inequality, neighbourhood affluence and population health. Soc Sci Med 1982. 2005;60(7):1557–69. 10.1016/j.socscimed.2004.08.033 15652687

[43] Wilkinson RG. Mortality and distribution of income. Low relative income affects mortality. BMJ. 1998;316(7144):1611–2. 9616035

[44] Sergeant JC, Firth D. Relative index of inequality: definition, estimation, and inference. Biostatistics. 2006;7(2):213–24. 16192414 10.1093/biostatistics/kxj002

