# Correction: Are socio-economic inequalities related to cardiovascular disease risk? A systematic review and meta-analysis of prospective studies

**DOI:** 10.1186/s12872-024-04418-5

**Published:** 2025-01-10

**Authors:** Ololade J. Baruwa, Federica Alberti, Sunday Onagbiye, Annalisa Guddemi, Anna Odone, Hannah Ricci, Maddalena Gaeta, Schmid Daniela, Cristian Ricci

**Affiliations:** 1 Africa Unit for Transdisciplinary Health Research (AUTHeR), North-West University, Potchefstroom, South Africa; 2 University of Cape Town, Cape Town, South Africa; 3 Department of Public Health, Experimental and Forensic Medicine, University of Pavia, Pavia, Italy; 4 Health & Exercise Science, Frederick Community College, Frederick, MD USA; 5 Faculty of Life Sciences, Albstadt-Sigmaringen University, Sigmaringen, Germany

**Correction: BMC Cardiovasc Disord 24, 685 (2024)**

**https://doi.org/10.1186/s12872-024-04248-5**

In the original publication of this article [1], the given and family names of Ololade J. Baruwa were inadvertently structured. The incorrect and correct names are presented with this correction article. The original article has been updated.

Incorrect author name: Julius B. Ololade

Correct author name: Ololade J. Baruwa
