# Supplementary material for: Are socio-economic inequalities related to cardiovascular disease risk? A systematic review and meta-analysis of prospective studies
Source: BMC Cardiovasc Disord. 2024 Nov 27;24:685. doi: 10.1186/s12872-024-04248-5 (PMC11603974; doi:10.1186/s12872-024-04248-5)
Supplement: Supplementary file 3 — Supplementary Material 3 [file 12872_2024_4248_MOESM3_ESM.pdf]

((Adult [Mesh]) OR (adult [Title/Abstract]) OR (adulthood [Title/Abstract]) AND (social status [Title/Abstract]) OR (factors, socioeconomic[MeSH Terms]) OR (socioeconomic status[MeSH Terms]) OR (condition, economic[MeSH Terms]) AND ((cardiovascular system [MeSH Terms]) OR (cardiovascular system [Title/Abstract]) OR (cardiovascular diseases [Title/Abstract]) OR (cardiovascular diseases [MeSH Terms]) NOT (mortality[Title/Abstract]) NOT (hypertension[MeSH Terms]) NOT (hypertension[Title/Abstract]) NOT (risk factor[MeSH Terms]) NOT (risk factor [Title/Abstract]) NOT (mortality[MeSH Terms])) AND ((cohort studies[MeSH Terms]) OR (cohort studies[Title/Abstract]) NOT (survey[Title/Abstract]) NOT (retrospective[Title/Abstract]))
